# Validity and reliability of the Turkish version of the Cold Intolerance Symptom Severity Questionnaire

**DOI:** 10.3906/sag-1808-170

**Published:** 2019-08-08

**Authors:** Nurten Gizem TÖRE, Mesken GÜMÜŞSOY, Deran OSKAY

**Affiliations:** 1 Department of Physiotherapy and Rehabilitation, Faculty of Health Sciences, Gazi University, Ankara Turkey; 2 Department of Physiotherapy and Rehabilitation, Özel Deva Hospital, Ankara Turkey

**Keywords:** Cold intolerance, hand injury, Turkish version, validity-reliability

## Abstract

**Background/aim:**

The aim of this study was to determine validity and reliability of the Turkish version of the Cold Intolerance Symptom Severity (CISS-T) Questionnaire.

**Materials and methods:**

The translation and back translation steps of the study were based on the Beaton guidelines. Sixty-eight patients between 18 and 65 years old with cold intolerance after amputation, replantation, multiple crush syndrome, and peripheral nerve injury were included in the study. Patients completed the Disabilities of the Arm, Shoulder, and Hand Questionnaire (DASH), the SF-36 Quality of Life Questionnaire, and the single questions assessing the cold sensitivity and cold intolerance once and the final version of the CISS-T twice with a 7-day interval.

**Results:**

The internal consistency (Cronbach α = 0.844) and test-retest reliability (r = 0.938) of CISS-T were assessed and both were considerably high. Also, the correlations between the scores of the CISS-T, DASH-T, SF-36-T, and the single questions were analyzed by Spearman’s correlation coefficient. The CISS-T showed an excellent correlation with the single questions (rho = 0.8 and 0.877), a good and negative correlation with the pain subscale of the SF-36 (rho = 0.617), and a moderate correlation with the DASH-T (rho = 0.592).

**Conclusion:**

As a result, the CISS-T is a valid and reliable instrument to assess the severity of cold intolerance.

## 1. Introduction

Cold intolerance is defined as an abnormal or extreme reaction that occurs in the hands and fingers following exposure to cold in peripheral nerve injuries. This reaction is accompanied by pain, discoloration, stiffness, weakness, and numbness in the extremities (1,2). In addition to the feeling of discomfort, the individual avoids and takes precautions against exposure of injured extremities to cold (3). Cold intolerance is frequently observed after soft tissue, nerve, arterial, and bone injuries of the upper extremity (4,5). It is also seen in workers who are exposed to repetitive vibration (6).

The incidence of cold intolerance is high in upper limb nerve injuries. Cold intolerance is the most disturbing, long-lasting symptom observed in the vast majority of patients with peripheral nerve injury, and it affects both occupational and leisure activities (7–9). Symptoms of cold intolerance do not occur immediately after the injury (10). Typically, it develops in the first 4 months after the injury and reaches the highest level 1 year after the injury (11). Therefore, the assessment of cold intolerance is significant for the management of patients (12).

Cold intolerance can be evaluated by objective and subjective methods (13,14). In a pilot study published by Ruijs et al., infrared thermography was reported to be a tool that could be used to assess the distribution of cold intolerance objectively (13). The devices used to evaluate cold intolerance objectively may not always be available. Questionnaires or tests can be used to assess cold intolerance subjectively (14). They are easy to reach and also universally accepted as outcome measurement instruments. They are used in clinical trials frequently since they are practical and less time-consuming. These tools are helpful in detecting the health and disability problems of the patient (15). In the literature, various tests have been used, such as visual analogue scale (VAS), single questions questioning the presence, absence, or severity of the pathology, or questionnaires consisting of multiple questions evaluating different aspects of the pathology in the subjective evaluation of cold intolerance (16). 

The questionnaire was originally developed in 1991 by McCabe et al. as 4 questions. It was later modified by Irwin et al. in 1997 as the Cold Intolerance Symptom Severity scale. The modified CISS questionnaire contains 6 questions that investigate the symptoms, frequency, duration, and severity of cold intolerance and its effects on daily living activities (14). 

The CISS was designed to ensure a more discriminating indication of severity in a wider range of symptom intensity, reflecting the severity of symptoms developed by 4 McCabe scenarios. In addition, it defined more relevant symptom-specific features such as the persistence and frequency of symptoms and how these disrupted daily living activities (14). The CISS combines all the different characteristics of previously developed methods to evaluate the severity of cold intolerance. Therefore, the CISS allows a more comprehensive assessment of cold intolerance in many different aspects (14,17). The CISS has been described as the most clear and comprehensive questionnaire available to assess cold intolerance in the literature (18). These properties make CISS important for the clinical use to assess the severity of cold intolerance.

The validity and reliability study of the Swedish version of the CISS questionnaire was conducted by Carlsson et al. in 2007 (12). However, there is no Turkish version of such a comprehensive questionnaire evaluating the severity of cold intolerance. Therefore, the purpose of this study was to assess the suitability of the CISS-T for the Turkish society and the effectiveness of its clinical use.

## 2. Materials and methods

This study was composed of two phases: the first one was the translation and cross-cultural adaptation of the CISS to the Turkish language, and the second one was the validity and reliability testing for the CISS-T. 

### 2.1. Translation and cross-cultural adaptation

First of all, Irwin, the developer of the CISS, granted authority to perform the Turkish translation of the CISS. Then, the cross-cultural adaptation process of the CISS was performed according to Beaton’s guidelines, which consisted of five stages (19). Firstly, the English version of the CISS was translated into Turkish by two bilingual, native Turkish people, one physiotherapist, and one English linguist with no medical background. Secondly, two different translations of the CISS were merged into a single Turkish version. Immediately after this step, two professional bilingual translators retranslated the merged version of the CISS into English. Afterwards, the translation was reviewed in terms of its cultural and linguistic quality by a committee which was composed of forward and backward translators, a methodologist, and a Turkish linguist. Some slight changes were made to perform the cultural adaptation. In the final step, the prefinal Turkish version of the CISS was field tested on 20 Turkish patients with cold intolerance. When the patients stated that they understood each question clearly, the questionnaire was finalized.

### 2.2. Patients

The study included 68 patients with cold intolerance after amputation, replantation, multiple crush syndrome, and peripheral nerve injury (20). In this study, all patients were treated as one group as in the Swedish version of the study (12). The patients were referred to the outpatient department of physiotherapy and rehabilitation at Gazi University, the Faculty of Health Sciences. All the patients gave their signed consent before the interview. This research was approved by the Ethics Committee of Gazi University Health Sciences Institute. The data were gathered between February 2017 and April 2018. One of the researchers gave an explanation to the patients about cold intolerance and asked them if they suffered from any symptoms of cold intolerance on exposure to cold. If the answer was affirmative, they were included in the study as in the Swedish version of the study (12). The other inclusion criteria were being between 18 and 65 years of age and being literate in Turkish. Nonnative Turkish patients and patients who were not willing to participate were excluded from the study. 

### 2.3. Instruments

The Disabilities of Arm, Shoulder, and Hand (DASH) questionnaire is an upper extremity-specific outcome measure. It is a valid and reliable patient-reported outcome among patients with upper extremity disorders. The DASH consists of 30 items to assess disorders about upper extremity. It includes questions about pain, stiffness, weakness, vocational functions, ability to perform daily activities (dressing, eating, sleeping, etc.), family, and self-care. The DASH scores range from 0 to 100 (21). Higher DASH scores indicate more severe disability cases. The Turkish translation and the cross-cultural adaptation of the DASH were performed by Düger et al. (22).

The Short Form-36 (SF-36) quality of life scale was developed by the Rand Corporation in 1992 to assess health-related quality of life (23). The SF-36 questionnaire is a self-administered questionnaire containing 36 items. It measures health on 8 multiitem dimensions, covering functional status, well-being, and overall evaluation of health (24). The SF-36 is a comprehensive scale that measures physical function and related skills. However, the fact that it does not assess sexual function is a limitation of this scale. It evaluates the impact of health conditions on the lives of patients over the past 1 month (4 weeks) (25). The score of each subscale is calculated separately (24). This scale, which has translations in different languages, has been used to assess health status in various disease groups. Koçyiğit et al. conducted the validity and reliability study of the Turkish version of the SF-36 in 1999 (26). 

Different instruments have been used in the literature to assess the severity of cold intolerance. However, these instruments cannot assess cold intolerance extensively. Thus, in 1997, Irwin et al. developed the Blond McIndoe Cold Intolerance Symptom Severity (CISS) questionnaire, which allows for a more global assessment of cold intolerance by combining the features of the previous methods (14). It addresses the severity of cold intolerance in greater detail than a single yes or no question. It consists of 6 questions in total. However, the first question is not included in the score. This question inquires the symptoms of cold intolerance including pain, numbness, stiffness, weakness, swelling, and skin color change. Other questions concern the frequency, duration, and severity of cold intolerance, and the effects of the symptoms of cold intolerance on daily living activities (17). Similar to the VAS, a score between 0 and 10 is assigned for questions 1 to 5. In these questions, 0 means no symptoms, whereas 10 means the most severe symptoms. Question 6 is evaluated between 0 and 4 points. The subscales of this question are answered by ticking. When all the questions are answered, a CISS score of minimum 4 and maximum 100 points is obtained (12,14). CISS scores have been grouped into 4 classes as extremely severe (76–100), severe (51–75), moderate (26–50), and mild (4–25) cold intolerance (14). The CISS questionnaire has been validated in the patient group with peripheral nerve injury (14). The validity and reliability study of the Swedish version of the CISS questionnaire developed in English was conducted by Carlsson et al. in 2007 (12). There is no Turkish questionnaire evaluating the severity and frequency of cold intolerance and its effects on daily living activities.

### 2.4. Analysis of reliability and validity

Reliability is a measure of consistency. Internal consistency was analyzed using Cronbach’s alpha. Cronbach’s alpha value indicates the internal correlation of all the items in a questionnaire. A Cronbach’s alpha value that is greater than 0.70 is considered to demonstrate relevant internal consistency. Reliability was also tested using the test-retest method. Accordingly, it was assessed by administering the CISS to the same patients with an interval of 7 days. The correlation between the total scores of both tests was analyzed using Spearman’s correlation coefficient. A correlation coefficient (rho) of 1 shows a perfect correlation, whereas 0 indicates no reproducibility (27). 

Validity is an indicator of how well a questionnaire measures what it is supposed to assess. The construct validity was tested by comparing the total results of the CISS with the DASH, the SF-36, and single questions about cold intolerance. Spearman’s correlation coefficient was used to evaluate the association between the questionnaires. Spearman’s correlation coefficients ranging between 0.81 and 1.00 were considered excellent, while 0.61 and 0.80, 0.41 and 0.60, 0.21 and 0.40, and 0 and 0.20 were accepted as very good, good, weak, and bad, respectively (27). 

### 2.5. Statistical analysis

Statistical analysis of the data was performed using SPSS version 22.0 (IBM). Statistical data were stated as mean ± standard deviation (X ± SD), median, or percent (%). The single-sample Kolmogorov–Smirnov test was used to demonstrate the parametric or nonparametric distribution of the data. The analyses of test-retest and internal consistency were performed in order to determine the reliability of the CISS questionnaire. The retest method of the form is used in the analysis of reliability in cases where it is possible to regain access to the same group of samples earlier in the study. The CISS-T, which was applied to 68 people in their first visit, was applied to the same 68 people in their second visit. Test-retest reliability was tested using Spearman’s *ρ* correlation coefficients. The internal consistency of the CISS was analyzed using Cronbach’s *α *coefficient. A Cronbach’s alpha coefficient greater than 0.70 was considered significant. The construct validity of the CISS was tested by correlating total scores of the CISS with total scores of the DASH-T, the pain subscale of the SF-36, and single questions about cold intolerance by using Spearman’s *ρ* correlation coefficients. Statistical significance was accepted as P < 0.05 (27,28). 

## 3. Results

### 3.1. Demographic information

The study included 68 patients (17 females, 51 males; mean age: 38.38 ± 11.23; age range: 18 to 65) with cold intolerance. The average age of the females was 32.88 ± 8.15 (min 21, max 44) while the average age of the males was 40.22 ± 11.58 (min 18, max 65). Most of the patients included in the study were male, their education level was high school, their dominant side was right, and their affected side was left (Table 1). 

**Table 1:** Numbers and percentages of information about patients’ sex, educational level, dominant, and affected extremity, and diagnoses.

	n	%
SexWomanMan	1751	2575
Education levelPrimary schoolMiddle schoolHigh schoolUndergraduateGraduate	15824183	22.111.835.326.54.4
Dominant handRightLeft	5216	76.523.5
Affected handRightLeft	2444	35.364.7
DiagnosisAmputationReplantationDigital nerve injuryRadial nerve injuryMedian nerve injuryUlnar nerve injuryCombined injury of median and ulnar nerveBrachial plexus injuryMultiple crush injury	15727357121	22.110.339.74.47.410.31.52.91.5

When the diagnosis of the patients participating in the study was examined, it was seen that most of the patients had digital nerve injury. The least common diagnoses were multiple crush syndrome and combined median and ulnar nerve injuries (Table 1).

The median values and the interquartile ranges (IQR) for the test and retest score of the CISS-T were 44 (22–55) and 42.5 (25–53), respectively. These results indicate that patients had moderate cold intolerance (Table 2).

**Table 2:** Median value of test and retest scores of the CISS-T.

	M(IQR)	Min–max
CISS-T test score	44 (25–55)	10–95
CISS-T retest score	42.5 (25–53)	11–83

Min: minimum, Max: maximum.

### 3.2. Difficulties in the cultural adaptation

Moving between different languages is challenging. The translation process should lead to minimization of inconsistencies and errors in terms of criteria, content, techniques, meaning, or concepts (29). Standardized methods are of importance to compare groups from different cultures and nations and the questionnaires must be adapted to the intended culture to intensify validity (30,31). Providing interlanguage adaptations has an important place in the international use of questionnaires. This requires a highly effective methodology for the translation process (31). In this study, a question was found to be incompatible with the answers. In addition, patients stated that they could not understand the scoring system of some questions. Thus, in order to increase the intelligibility of the CISS, certain changes were made until all 20 individuals involved in the pilot study understood all the questions.

### 3.3. Reliability analysis of the CISS questionnaire

Cronbach’s alpha value was used for the internal consistency analysis of the CISS questionnaire. This value was found to be 0.844 for the entire questionnaire. This indicates that the internal consistency of the questionnaire is high. When each question was excluded, the Cronbach’s alpha value varied from 0.821 to 0.861 (Table 3). 

**Table 3:** Alpha coefficients with the exclusion of the item and the total Alpha of the dimensions.

Questions	Cronbach’s alpha value
If 2. Question excluded	0.830
If 3. Question excluded	0.836
If 4. Question excluded	0.845
If 5a. Question excluded	0.861
If 5b. Question excluded	0.821
If 5c. Question excluded	0.828
If 5d. Question excluded	0.842
If 5e. Question excluded	0.825
If 6a. Question excluded	0.838
If 6b. Question excluded	0.842
If 6c. Question excluded	0.842
If 6d. Question excluded	0.841
If 6e. Question excluded	0.838
Total	0.844

The data obtained were statistically significant at the 1% significance level (P < 0.001). When the correlation coefficients between two visits were examined, the rho value was found as 0.938. This value indicates that the questionnaire is unchanged over time. 

### 3.4. Validity analysis of the CISS questionnaire

One of the methods used to test the validity of a questionnaire is hypothesis test. The score obtained from the CISS was inversely proportional to the pain subscale of the SF-36. However, it was determined that the score obtained from the CISS Questionnaire was positively proportional to the DASH questionnaire and the single questions which evaluate cold sensitivity and cold intolerance. According to the results of the statistical analysis, it was found that the CISS questionnaire had a positive****and good correlation with the single questions that assessed cold sensitivity (r: 0.800) and cold intolerance (r: 0.877), a negative and good correlation with the pain subscale of the SF-36 (r: −0.617), and a positive and moderate correlation with the DASH-T Questionnaire (r: 0.592) (Table 4).

**Table 4:** Comparison of the CISS questionnaire with the DASH, the SF-36, and single questions.

CISS		
	r	P
First single question on sensitivity to cold	0.800	<0.001
Second single question on cold-intolerance	0.877	<0.001
SF-36 pain subscale	−0.617	<0.001
DASH-T	0.592	<0.001

## 4. Discussion

Cold intolerance is a common symptom developing after upper extremity injuries which adversely affects health-related quality of life (4). Although there are several other questionnaires evaluating cold intolerance, as mentioned before, the CISS evaluates cold intolerance in many different ways and in a more comprehensive way, taking into account the different characteristics of previously developed methods (14). Therefore, it is defined as the most clear and comprehensive questionnaire evaluating cold intolerance in the literature (18).

### 4.1. Translation and cultural adaptation

In our study, the criteria specified by Beaton et al. were used for translation from English into Turkish (19). Some difficulties arose during the translation, as in the Swedish version of the CISS. During the development of the Swedish version of the questionnaire, certain words and expressions could not be directly translated. However, they were arranged in a way that reflected the meaning of the original version of the questionnaire (12). After the first pilot implementation, we consulted the author of the original version of the CISS and made certain changes presented below that would not alter the core of the questionnaire. The question “Are the symptoms relieved?” was changed to “In how many minutes do these symptoms relax?” in order to ensure compliance with the answers in question 3. Patients had difficulty understanding the scoring system of questions 1, 5, and 6. Therefore, the necessary scales and explanations were added to these questions. At the end of questions 1 and 5, the statement “give a value between 0 and 10” and a 10-cm VAS were added. After the first 5 questions, the scoring system was changed in question 6. Patients found this change confusing. Hence, in order to increase intelligibility, the scores were given in a table. We think that the final version of the CISS-T is better understood and answered by the Turkish-speaking community.

### 4.2. Reliability and validity

The reliability and validity of the CISS were determined. The CISS indicated good internal consistency and very strong test-retest reliability. According to the analyses, all Spearman’s correlation coefficients were statistically significant and the results ranged from moderate to very strong. It can be clearly concluded that the CISS was successfully translated into Turkish and cross-culturally adapted and it is found to be a valid and reliable questionnaire to assess cold intolerance in Turkish-speaking patients.

The Cronbach’s alpha value for the CISS was 0.844, which demonstrates a good internal consistency. The Cronbach’s alpha coefficient is slightly higher in the Swedish version of the CISS (0.91) (12). We consider high Cronbach’s alpha values may indicate that the questions of the questionnaire are complementary and may correctly question the pathological findings. 

In the Swedish and the original version of the questionnaire, the Cronbach’s alpha value, which was generated when each item was deleted, was not analyzed (12,14). In this study, when each item was deleted, the Cronbach’s alpha value varied from 0.821 to 0.861. The CISS contains fewer questions compared to other questionnaires and all the questions are directly related to the symptoms and effects of the disease. These might be the reasons for close Cronbach’s alpha values when an item is omitted.

The test-retest interval of the present study was determined based on the study by Marx et al. (32). According to the results of this study, there was no statistical difference between test-retest results of implementations at 2 days or 2 weeks. When the literature is reviewed, there is no exact time interval for the test-retest method. The short duration between the tests may increase the likelihood of patients remembering the questions. This may lead to higher analysis results. The time interval was 6 months for the original version and 1 month for the study of the Swedish version conducted by Carlsson et al.. In our study, the time interval for the test-retest of the CISS Questionnaire was determined to be 7 days. Based on the study of Marx et al., we think that this time interval is suitable for test-retest reliability and does not affect the results of our statistical analysis. Spearman’s correlation coefficient used for the test-retest analysis of our study was found to be 0.938 as the resultant rho value. Irwin et al. reported a correlation coefficient of 0.90 (14). In the Swedish version of the questionnaire, Carlsson et al. found an interclass correlation coefficient of 0.92 in the test-retest method analysis (12). The CISS-T had a statistically proven high level of invariance with respect to time.

Irwin et al. did not perform validity analyses when developing the CISS questionnaire (14). The Swedish version of the questionnaire used hypothesis testing to determine validity. Spearman’s correlation in their results showed correlation coefficients between the CISS and the DASH, the CISS and the pain subscale of the SF-36, the CISS and the first single question, and the CISS and the second single question. These correlation coefficients were found as 0.73, −0.640, 0.730, and 0.810, respectively (12). In the present study, Spearman’s correlation coefficients between the CISS-T and the DASH-T, the CISS-T and the pain subscale of the SF-36, the CISS-T and the first single question, and the CISS-T and the second single question were 0.592, −0.617, 0.80, and 0.877, respectively. Although the DASH contains a number of questions about the symptoms of cold intolerance, it generally examines whether the patient can perform activities involving the entire upper limb or not and determines the level of disability. However, the pain subscale of the SF-36 and single questions were directly associated with the symptoms of cold intolerance. Therefore, this might be a reason for lower correlation between the CISS and DASH questionnaires. The evaluation of the results of Spearman’s correlation analysis revealed that the CISS-T is a valid instrument.

Low-reliability questionnaires lay the ground for bias in the measurement, and in particular some erroneous decisions in clinical practice. Therefore, the reliability of the questionnaires used must be well known. In fact, the high reliability of the tests used is an important requirement in the field of health (33). The CISS is a useful questionnaire for monitoring changes that may occur over time in the severity of symptoms of cold intolerance, a prognostic indicator in the clinic (12). The study of the Turkish version of the CISS questionnaire plays an important role in terms of comprehensively evaluating the severity of cold intolerance in the Turkish population. Besides, it is important to find that the questionnaire is reliable and valid at the end of the study for its use in clinical researches.

To the best of our knowledge, there is no reliable and valid Turkish outcome measure for the assessment of the severity of cold intolerance. In this study, we demonstrated that the CISS, which was successfully translated and cross-culturally adapted, is a reliable and valid questionnaire to assess the severity of cold intolerance. It can be used as a tool for evaluation both before and after the treatment to determine the severity of cold intolerance in Turkish patients. Furthermore, using this Turkish version, it can be possible to compare the results obtained from Turkish patients who have cold intolerance with international data.

Cold intolerance can also be observed in certain chronic diseases. Therefore, validation studies of the CISS for specific chronic diseases may be helpful and suggested for future studies.
